# Relation of Urine Cell-Free Mitochondrial DNA Quantified by Droplet Digital Polymerase Chain Reaction With Tubule Injury, Dysfunction, and Ambulatory Acute Kidney Injury

**DOI:** 10.1016/j.xkme.2026.101257

**Published:** 2026-01-08

**Authors:** Avnee J. Kumar, Katharine E. Epler, Joachim H. Ix, Vasantha Jotwani, Ronit Katz, Michael G. Shlipak, Mark L. Hepokoski

**Affiliations:** 1 Division of Pulmonary and Critical Care and Sleep Medicine, University of California San Diego, San Diego, CA; 2 Veterans Affairs San Diego Healthcare System, San Diego, CA; 3 Division of Nephrology-Hypertension, Department of Medicine, University of California San Diego, San Diego, CA; 4 Kidney Health Research Collaborative, Department of Medicine, San Francisco Veterans Affairs Health Care System and University of California San Francisco, San Francisco, CA; 5 Department of Obstetrics and Gynecology, University of Washington School of Medicine, Seattle, WA

To the Editor:

Early recognition of patients who will develop worsening kidney function is challenging. The kidney is dependent on a large pool of mitochondria to maintain its high metabolic rate, and mitochondrial dysfunction is a pathophysiological feature of both acute kidney injury (AKI) and chronic kidney disease (CKD).^1^^,^^2^ During mitochondrial stress, mitochondria release mitochondrial DNA (mtDNA) into the extracellular space, and cell-free mtDNA (cf-mtDNA) concentrations have been associated with kidney mitochondrial dysfunction.^3^^,^^4^ However, translational studies focused on urine cf-mtDNA have been limited by challenges in accurate and reproducible methods of quantification. For example, prior studies have used real-time polymerase chain reaction (RT-PCR) targeting one of the 37 mitochondrial genes, but there has been variability in results depending on the sequence targeted.^5^ RT-PCR also requires a DNA isolation step and generation of a standard curve, which introduce additional sources of error. Recently, we used next-generation sequencing to identify the optimal target sequence for cf-mtDNA and published novel methods of quantification in plasma using droplet digital PCR (ddPCR).^6^ Importantly, our methods provide an absolute cf-mtDNA concentration without a DNA isolation step. To extend beyond plasma measurement, in this study, we evaluated the feasibility of our methods for urine cf-mtDNA quantification in the ambulatory setting. We explored the relationship between urinary cf-mtDNA and established biomarkers of kidney tubule health and evaluated whether cf-mtDNA is associated with risk of future AKI.

In this nested case-control study of participants with CKD from the Systolic blood PRessure Intervention Trial, we identified individuals who developed ambulatory AKI at follow-up visits (cases, ≥0.3 mg/dL increase in serum creatinine from baseline to visits at 12 or 24 months), and 1:1 matched controls based on age ± 5 years, baseline estimated glomerular filtration rate (eGFR) ± 5, and sex (Item S1, Table S1).^7^ First, we asked whether urine cf-mtDNA concentrations correlate with known measures of kidney tubule health. Second, we asked whether baseline urine cf-mtDNA concentrations were associated with ambulatory AKI. We hypothesized that urinary cf-mtDNA quantified using ddPCR would positively associate with markers of proximal tubule injury and subsequent ambulatory AKI. All participants provided written informed consent, and the study was approved by the institutional review board of each participating site.

In this pilot study, we conducted mtDNA measures only in persons with CKD at baseline (eGFR < 60 mL/min/1.73 m^2^), as we had made urine tubule biomarkers in this subset previously. Thus, we identified 103 AKI cases (n = 103) who were matched to 103 controls (Table S2). Spot urine specimens were obtained and frozen at –80 °C until measurement. No centrifugation, preservatives, or protease inhibitors were used during initial processing. We then quantified cf-mtDNA in urine using ddPCR targeting a small sequence in the mitochondrial NADH dehydrogenase-1 (ND1) gene as shown previously in plasma.^6^ This analysis enabled the absolute quantification of target mtDNA sequences, with the concentration of the sequence calculated based on the fraction of positive droplets. Urine cf-mtDNA was reported in copies per 20 μL and all measurements were completed in duplicate. Next, we evaluated associations of urine cf-mtDNA with baseline clinical kidney measures of glomerular health (serum eGFR and albuminuria). Last, we evaluated associations of urine cf-mtDNA with urine biomarkers of proximal tubule reabsorption, proximal tubule injury, tubule inflammation, and tubular reserve as shown in Table 1. Of note, several of these markers have been associated with future ambulatory AKI.^7^ The outcome (urine cf-mtDNA) and exposure (biomarker) were log transformed for analysis. Relationships between urinary cf-mtDNA and markers of kidney injury were completed using linear regression with a case/control interaction completed with stratified analysis. All models were adjusted for age, sex, and urine creatinine.

**Table 1:** Association of Serum eGFR and Urine Biomarkers With cf-mtDNA in All Patients at Baseline Adjusted for Age, Sex, Race, and Urine Creatinine.

Function	Marker	Change in Marker per 1% Higher Urine cf-mtDNA
Glomerular function	Serum eGFR	–23.96 (–91.48 to 43.57)
	Urine albumin	11.0 (–1.0 to 23.1)
Reabsorbed by proximal tubule	Urine α1-microglobulin	**27.1 (6.4-47.8)**
	Urine β2-microglobulin	**8.6 (3.8-13.5)**
	Urine trefoil factor 3	**24.8 (14.8-34.9)**
Produced in injured proximal tubule cells	Urine neutrophil gelatinase-associated lipocalin	**10.9 (5.4-16.3)**
	Urine kidney injury molecule-1	**46.8 (23.1-70.4)**
	Urine clusterin	8.0 (–9.4 to 25.5)
Tubule inflammatory cell products and attractants	Urine monocyte chemoattractant protein-1	**38.3 (9.0-67.6)**
	Urine interleukin-18	17.3 (–4.7 to 39.3)
	Urine chitinase-3-like protein-1	**30.3 (18.4-42.2)**
Indicative of tubule reserve	Urine urodmodulin	14.0 (–16.5 to 44.5)
	Urine epidermal growth factor	7.2 (–28.6 to 43.1)

*Note*: % Increase/Decrease (95% CI) representing the percent change in cf-mtDNA associated with a 1% change in the marker. Significant changes in bold. cf-mtDNA and all markers were log transformed. Urine markers reported were stratified by primary function: albumin to represent the glomerular injury; α1-microglobulin, β2-microglobulin, and trefoil factor 3 markers reabsorbed by the proximal tubule; neutrophil gelatinase-associated lipocalin, kidney injury molecule-1, and clusterin markers produced in injured proximal tubule cells; monocyte chemoattractant protein 1, interleukin-18, and chitinase-3-like protein-1 as tubule inflammatory cell products and attractants; and uromodulin and epidermal growth factor indicating tubule reserve.

Abbreviations: CI, confidence interval.

Urine cf-mtDNA concentrations were in the detectable range for all 206 participants and correlated positively with concentrations of 7 urine tubule health biomarkers (Table 1). The median and interquartile range of baseline urine cf-mtDNA measured was 125 (57-262) copies/20 μL in control patients and 251 (80-543) copies/20 μL in ambulatory AKI patients. The lowest detected value for urine cf-mtDNA was 19.22 copies/20 μL. The coefficient of variation in duplicate samples had an average of 12.4%, and median of 7.8%. Associations were strong, with a 1% higher urine cf-mtDNA concentration associating with a 9%-46% higher concentration of urine biomarkers of tubule health. Associations appeared strongest with urine biomarkers of proximal tubule reabsorption, proximal tubule injury, and tubule inflammation. On the other hand, associations with glomerular makers (serum eGFR and urine albumin) were not significant. Finally, every doubling of baseline urine cf-mtDNA concentration was associated with 17% greater odds of developing subsequent ambulatory AKI (odds ratio, 1.17; 95% confidence interval, 1.0-1.35) (Fig 1). A limitation of our study is the definition of “ambulatory AKI” requiring changes in serum creatinine over 1-2 years, as opposed to over 48 hours or changes in urine output. Unlike inpatient AKI events, standardized definitions for AKI in the ambulatory setting are not available. To address this issue, we use the term “ambulatory AKI”, which is consistent with terminology used in our prior publication.^8^

**Figure 1:**
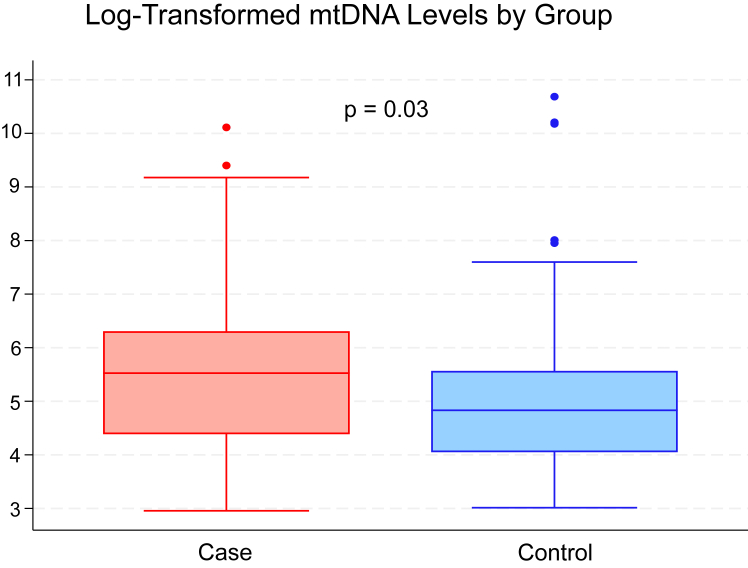
Log 2 transformed baseline levels of mitochondrial DNA in ambulatory AKI case patients versus control patients. Odds ratio 1.17 and 95% confidence interval (1.01-1.35), for every doubling of cf-DNA, the odds of having ambulatory AKI increase by 17%. Corrected for age, sex, race, and urine creatinine.

These results demonstrate that measurement of urine cf-mtDNA is feasible using ddPCR without DNA isolation in an ambulatory population providing unprocessed spot urine specimens. Furthermore, urinary cf-mtDNA concentrations are strongly associated with other biomarkers of kidney tubule damage and subsequent risk of ambulatory AKI.

## Supplementary Materials

Supplementary File (PDF) Item S1; Tables S1-S2.

## References

[1] Hepokoski M., Wang J., Li K. (2021). Altered lung metabolism and mitochondrial DAMPs in lung injury due to acute kidney injury. Am J Physiol Lung Cell Mol Physiol.

[2] Jotwani V., Thiessen-Philbrook H., Arking D.E. (2025). Association of blood mitochondrial dna copy number with risk of acute kidney injury after cardiac surgery. Am J Kidney Dis.

[3] Whitaker R.M., Stallons L.J., Kneff J.E. (2015). Urinary mitochondrial DNA is a biomarker of mitochondrial disruption and renal dysfunction in acute kidney injury. Kidney Int.

[4] Eirin A., Herrmann S.M., Saad A. (2019). Urinary mitochondrial DNA copy number identifies renal mitochondrial injury in renovascular hypertensive patients undergoing renal revascularization: a pilot study. Acta Physiol (Oxf).

[5] Lubkin D.T., Bishawi M., Barbas A.S., Brennan T.V., Kirk A.D. (2018). Extracellular mitochondrial DNA and N-formyl peptides in trauma and critical illness: a systematic review. Crit Care Med.

[6] Hepokoski M.L., Odish M., Lam M.T. (2022). Absolute quantification of plasma mitochondrial DNA by droplet digital PCR marks COVID-19 severity over time during intensive care unit admissions. Am J Physiol Lung Cell Mol Physiol.

[7] Bullen A.L., Katz R., Lee A.K. (2019). The SPRINT trial suggests that markers of tubule cell function in the urine associate with risk of subsequent acute kidney injury while injury markers elevate after the injury. Kidney Int.

[8] Ascher S.B., Katz R., Estrella M.M. (2025). Associations of urine biomarkers during ambulatory acute kidney injury with subsequent recovery in kidney function: findings from the SPRINT study. Am J Kidney Dis.

