# Supplementary material for: Relation of Urine Cell-Free Mitochondrial DNA Quantified by Droplet Digital Polymerase Chain Reaction With Tubule Injury, Dysfunction, and Ambulatory Acute Kidney Injury
Source: Kidney Med. 2026 Jan 8;8(3):101257. doi: 10.1016/j.xkme.2026.101257 (PMC12936929; doi:10.1016/j.xkme.2026.101257)
Supplement: Supplementary File (PDF) — Item S1; Tables S1-S2. [file mmc1.pdf]

Item S1. *Study Design and Population*

Out of 652 eligible patients, excluding the intervention arm of the trial and those without baseline CKD, there were 104 remaining, 103 of which were successfully matched. Patients without CKD or ambulatory AKI were excluded due to lack of urine biomarker measurement. Out of 9361 eligible patients, excluding the intervention arm, overlapping cases, those with missing data, and those without baseline CKD, there was a pool of 3318 to interrogate as a controls (**Table S1**). The present study was conducted in accordance with the Declaration of Helsinki.

cf-mtDNA Methods

cf-mtDNA quantification was determined via ddPCR and completed in duplicate. The ddPCR reaction was set up in a total volume of 20 µL, comprising 10 µL of 2x ddPCR supermix for probes, 1 µL of 20x primer/probe mix (yielding final concentrations of 900 nM for each primer and 250 nM for the probe), 1 µL of urine sample, and 8 µL of nuclease-free water. No DNA extraction step was performed. The plate was placed into a droplet generator machine (Bio-Rad Laboratories, Hercules, CA), which generated droplets in an oil emulsification in a 96-well plate. The plate was then placed in an Applied Biosystems Veriti 96-well Thermal Cycler (Applied Biosystems, Waltham, MA) per manufacturer instructions. After cycling, droplets were analyzed with a QX200 droplet reader (Bio-Rad Laboratories, Hercules, CA), and data were processed using QuantSoft version 1.7.4 software, which distinguished between PCR-positive and PCR-negative droplets. Comparisons between cases and controls were completed using conditional logistic regression. Analyses were completed utilizing software StataCorp (2023, Release 18).

Table S1. CONSORT Diagram: Matched Case-Control Study

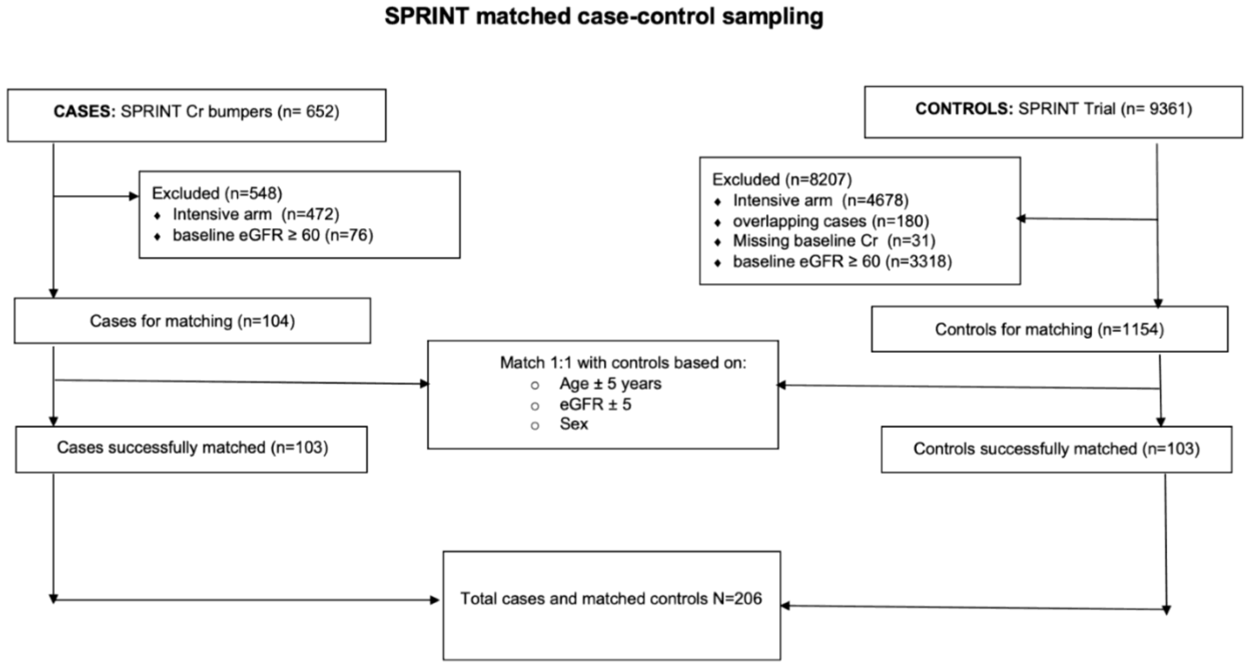

Table S2. Baseline characteristics of SPRINT participants with increase in serum creatinine of ≥ 0.3 mg/dL and matched controls. SD = standard deviation IQR = interquartile range

Baseline Characteristic	Controls (N=103)	Cases (N=103)	Total (N=206)
Age – no. (SD)	73 (10)	73 (10)	73 (10)
Female - no. (%)	41 (40)	41 (40)	82 (40)
Race - no. (%)			
White	62 (60)	53 (52)	115 (56)
African American	31 (30)	39 (38)	70 (34)
Hispanic	8 (8)	10 (10)	18 (9)
Other	2 (2)	1 (1)	3 (2)
Smoking - no. (%)			
Never	43 (42)	43 (42)	86 (42)
Former	51 (50)	41 (40)	92 (45)
Current	9 (9)	19 (18)	28 (14)
History of CVD - no. (%)	19 (18)	32 (31)	51 (25)
Systolic BP - mmHg (SD)	141 (17)	145 (17)	143 (17)
Diastolic BP - mmHg (SD)	75 (13)	75 (14)	75 (13)
Antihypertensive meds – no. (SD)	2.38 (1.05)	2.49 (1.07)	2.43 (1.06)
0	2 (2)	4 (4)	6 (3)
1	22 (21)	14 (14)	36 (18)
2	29 (28)	32 (31)	61 (30)
3	36 (35)	34 (33)	70 (34)
≥4	14 (14)	19 (18)	33 (16)
Antihypertensive class – no. (SD)			
Beta blocker	51 (50)	63 (61)	114 (55)
Diuretic	63 (61)	59 (57)	122 (59)
Calcium channel blocker	40 (39)	55 (53)	95 (46)
ACE inhibitor or ARB	77 (75)	62 (60)	139 (68)
BMI - kg/m^2 (SD)	30.1 (5.9)	30.0 (6.6)	30.0 (6.3)
Serum creatinine - mg/dL (IQR)	1.57 [1.27, 1.95]	1.57 [1.32, 1.88]	1.57 [1.30, 1.91]
eGFR - ml/min/1.73m^2 (IQR)	39 [31, 46]	38 [31, 46]	39 [31, 46]
Urine ACR - mg/g (IQR)	26 [9, 120]	109 [19, 268]	44 [13, 186]
